# Quantification of total and unbound cefuroxime in plasma by ultra‐performance liquid chromatography tandem mass spectrometry in a cohort of critically ill patients with hypoalbuminemia and renal failure

**DOI:** 10.1002/jcla.23100

**Published:** 2019-11-30

**Authors:** Joost J. van Raaij, Noortje J. D. Mabelis, Kimberly N. Shudofsky, Sjoerd D. Meenks, Jos L. M. L. le Noble, Paddy K. C. Janssen

**Affiliations:** ^1^ Department of Hospital Pharmacy VieCuri Medical Centre Venlo The Netherlands; ^2^ Department of Intensive Care VieCuri Medical Centre Venlo The Netherlands; ^3^ Department of Pharmacology and Toxicology Maastricht University The Netherlands; ^4^ Department of Clinical Pharmacy and Toxicology Maastricht University Medical Centre Maastricht The Netherlands

**Keywords:** cefuroxime, hypoalbuminemia, intensive care unit, therapeutic drug monitoring, unbound concentration

## Abstract

**Background:**

Pharmacokinetic studies of cefuroxime by ultra‐performance liquid chromatography tandem mass spectrometry (UPLC‐MS/MS) have been limited to measurements of total concentrations. Here, we developed a robust method for quantifying total and unbound cefuroxime concentrations using UPLC‐MS/MS and ultrafiltration in critically ill patients with hypoalbuminemia and renal failure.

**Methods:**

Method validation included accuracy, linearity, precision, repeatability, recovery, and limit of quantification (LOQ). Feasibility of the method was performed on samples obtained from randomly selected intensive care unit (ICU) patients. Total and unbound cefuroxime concentrations were quantified using UPLC‐MS/MS. Sampling times were categorized as trough (180‐1 min prior to administration), peak (10‐30 min after administration), mid (30‐360 min after administration), and continuous (sampling during administration). Pharmacokinetic/pharmacodynamic (PK/PD) targets were unbound cefuroxime concentrations above 4 times the minimum inhibitory concentration (32 mg/L).

**Results:**

Intra‐assay and inter‐assay precision was <3%. Recovery was 99.7%‐100.3%, and LOQ was 0.1 mg/L. We included 11 patients (median age 72 years (range 54‐77). Median albumin serum concentrations and eGFR were 19 g/L (range 11‐40 g/L) and 48 mL/min/1.73 m^2^ (range 7‐115 mL/min/1.73 m^2^), respectively. Median trough and mid concentrations of total cefuroxime were 22.27 mg/L (range 5.42‐54.03 mg/L) and 71.49 mg/L (range 53.87‐73.86 mg/L), and median unbound fraction was 75.42% (range 27.36%‐99.75%). Median unbound cefuroxime concentrations were 11.94 mg/L (range 3.85‐32.39 mg/L) (trough) and 55.62 mg/L (range 10.03‐62.62 mg/L) (mid).

**Conclusion:**

The method is precise and accurate according to ISO 15189 and within the clinical range of cefuroxime (0.5‐100 mg/L). The method was applied in ICU patients and is suitable for TDM on unbound cefuroxime concentrations.

## 1 INTRODUCTION

Dutch guidelines on the management of sepsis in the intensive care unit (ICU) recommend cefuroxime for empiric therapy in patients with community‐ or nosocomial‐acquired sepsis of unknown origin.^1^ Cefuroxime is a second‐generation cephalosporin antimicrobial drug with time‐dependent killing against gram‐negative and, to a lesser extent, gram‐positive bacteria. The effect of hypoalbuminemia for cefuroxime dosing in critically ill patients with low levels of albumin or renal failure is likely to have significant consequences on the drug's pharmacodynamics (PD) and pharmacokinetics (PK). Therapeutic drug monitoring (TDM)‐based dose optimization of cefuroxime could overcome the drug's pharmacokinetic variability, increase its target attainment, and prevent toxicity by overdosing.^2^, ^3^

Pharmacokinetic studies of cefuroxime have been limited to measurements of total concentrations.^4^, ^5^, ^6^ To our knowledge, only one study is published on the analysis of unbound concentrations of cefuroxime. However, analysis was performed by high‐performance liquid chromatography (HPLC) ultraviolet detection.^7^ In contrast, direct measurement of cefuroxime levels and unbound fractions using ultra‐performance liquid chromatography tandem mass spectrometry (UPLC‐MS/MS) has not been published before.^8^

Given the variability of drug protein binding in ICU patients with low albumin levels and renal failure, the main purpose of this study was to develop a reliable and sensitive method based on UPLC coupled with quadrupole‐linear ion trap MS/MS. Secondly, we applied the optimized method to plasma samples of ICU patients and assessed the extent of cefuroxime plasma protein binding for TDM‐based dose optimization.

## 2 MATERIALS AND METHODS

### 2.1 Chemicals and standards

Ammonium acetate of LC/MS quality was obtained from Sigma‐Aldrich. LC/MS‐grade methanol and 98%‐100% formic acid were purchased from VWR International. MilliQ water was produced in our hospital. Pasteurized plasma protein solution (GPO) plasma and fresh frozen plasma (FFP) were from Sanquin. Cefuroxime was purchased from Fresenius Kabi and cefazolin as internal standard was purchased from Eurocept.

### 2.2 UPLC‐MS/MS conditions and procedure

An Acquity H‐class UPLC system equipped with a BEH C18 50 × 2.1 mm 1.7 µm column, a Xevo^®^ TQD detector (Waters Corporation), and MassLynx^©^ software (Waters Corporation) was used. The mobile phase consisted of 2 mM ammonium acetate in water and 0.1% (v/v) formic acid (solution A) or 2 mM ammonium acetate in methanol with 0.1% (v/v) formic acid (solution B). A constant flow rate of 0.5 mL/min was used and a step‐wise gradient elution was started with 90% eluent A for 0.5 min, followed by 100% eluent B for 2.1 min, and ended with 90% eluent A for 2.4 min, for a total run time of 5 min. The column oven temperature was set at 40°C. Electrospray ionization MS/MS was used with the following settings: desolvation gas, 1000 L/h; cone gas, 50 L/h; desolvation temperature, 600°C; source temperature, 150°C; cone voltage, 30 V; and capillary voltage, 1.0 kV with collision energy 14 and 12 eV. Scan time was 0.5 s; interchannel delay was 0.003 s; inter‐scan delay was 0.02 s; MS inter‐scan was 0.003 s; and dwell time was 0.007 s at 1.6‐2.6 min after injection. Cefazolin and cefuroxime were fragmented in two daughter molecules. Mass transition (*m/z*) of cefazolin was recorded at 454.8 > 353 and 454.8 > 156; *m/z* of cefuroxime was recorded at 446.9 > 342.0 and 446.9 > 385.9.

### 2.3 Sample preparation and processing

Before injection into the UPLC system, all samples were processed as follows: 0.1 mL of the solution to be analyzed was taken and spiked with 30 µL cefazolin 0.05 mg/mL (as internal standard) and 500 µL methanol:acetonitrile 90%:10% (v/v). Patient samples were thawed and vortexed shortly before analysis and processed in the same manner. This mixture was vortexed for 1 min and ultracentrifuged at 30 000 *g* for 10 min at 25°C. Then, 2 µL of this sample was injected and quantified as described in Section 2.2.

### 2.4 UPLC‐MS/MS validation

Analysis validation was performed according to the International Standardization Organization (ISO) 15189:2012 guideline chapter 5.5.1.3.^9^ The clinical pharmaceutical laboratory is ISO 15189 accredited.

To determine the analysis’ specificity, a blank sample in GPO plasma was processed 10 times. Multiple reaction monitoring (MRM) transitions of the sample were compared to a standard containing 0.5 mg/L cefuroxime. To assess linearity, a calibration line was calculated using cefuroxime serial dilutions of 0.5, 5.0, 10, 25, 50, 75, and 100 mg/L in GPO plasma, and the correlation coefficient (*r*) was determined. Each standard was processed in three replicates. Reproducibility was tested by analyzing two control samples of the standard solution at 0.5 and 25 mg/L 10 times and further confirmed by testing the control samples ten times by two different analysts on two separate days. Recovery was determined by analyzing two standards of cefuroxime (0.5 and 25 mg/L) ten times using the method described in Section 2.2. Finally, the limit of quantification (LOQ) was estimated by analyzing the lowest standard (0.5 mg/L) at a twofold and fivefold dilution of 0.25 and 0.1 mg/L, respectively. Recovery was measured by UPLC‐MS/MS with ten injections for each diluted standard.

### 2.5 Plasma protein binding

A method to quantify cefuroxime fractions bound and unbound to plasma proteins was set up using three in vitro cefuroxime stock solutions at 2, 40, and 80 mg/L prepared in six replicates and diluted in FFP. These solutions were quantified as described in Section 2.2. Subsequently, unbound cefuroxime was quantified by pipetting 0.5 mL of the solution to be analyzed into a Centrifree^®^ Ultrafiltration Device (Merck Millipore). After centrifugation at 1500 *g* for 25 min at 25°C, 0.1 mL of the filtrate was processed and unbound cefuroxime was quantified as described in Section 2.2. Stability data (25°C for 25 minutes) were adopted from Hu and colleagues.^10^ The unbound fraction concentration was expressed as (total measured concentration – protein‐bound concentration)/ total measured concentration.

### 2.6 Study design and patients

This prospective, noninterventional feasibility study was conducted as a pilot study at VieCuri Medical Center, an in‐patient university‐associated teaching hospital in the province of Limburg, the Netherlands. The study protocol was approved by the medical ethical committee of Maastricht University Medical Centre (METC 17‐4‐025). A waiver for informed consent was granted, because samples were obtained from routine care procedures. Patient samples were collected between May 2017 and February 2018. Inclusion criteria encompassed patients aged ≥18 years who had received intravenous cefuroxime by intermittent or continuous infusion. Patients were excluded if they had received only one single infusion of cefuroxime during their stay on the ICU.

Patient demographics, clinical variables, antibiotic dosing of cefuroxime, and time of administration were retrieved from the patient data management system. Hypoalbuminemia was defined as a serum albumin level of <35 g/L.^11^ Intravenous dosing regimens were prescribed by the attending physician. Continuous infusion was performed with an automated pump system and intermittent dosing regimens were administered in 15‐30 min by an ICU nurse according to our local antibiotic treatment guideline. Standard cefuroxime regimen was 4500 mg/d in three doses by intermittent intravenous infusion, or 4500 mg/d by continuous infusion. Cefuroxime regimens were adjusted based on the estimated renal function (CKD‐EPI). Dosages were 1500 mg TID for patients with a glomerular filtration rate (eGFR) >30 mL/min/1.73 m^2^, 1500 mg BID for patients with eGFR of 10‐30 mL/min/1.73 m^2^, and 750 mg QD for patients with eGFR <10 mL/min/1.73 m^2^. Dialysis patients with intermittent hemodialysis (IHD) were treated with 750 mg BID, with the second administration following immediately after dialysis. Patients receiving continuous venovenous hemofiltration (CVVH) were given 750‐1500 mg BID.^12^

Leftover plasma samples were collected at room temperature (15‐25°C) in serum tubes as part of routine patient care. Samples were centrifuged after collection and frozen at −20°C for a maximum of six months to analysis.^10^ Samples were analyzed batch‐wise. Stability of plasma samples was not tested. Stability data of cefuroxime plasma samples were adopted from Hu and colleagues where corresponding storage times and temperatures (6 months at −20°C) were used.^10^

Time between sampling and exact drug administration times were calculated using our electronic administration registration. Sampling times were categorized as follows: trough (180‐1 min prior to administration), peak (10‐30 min after administration), mid (30‐360 min after administration), and continuous (sampling during continuous administration).

### 2.7 Statistical analysis

This study focused on the feasibility of the proposed method for routine clinical use in ICU patients, and therefore, no sample size calculation was deemed necessary.

Patient characteristics are presented as medians with ranges and interquartiles (IQR). Total and unbound plasma concentrations of cefuroxime are presented as medians with ranges and IQR and are visually displayed as box plots. Analytical values for in vitro validation of the UPLC‐MS/MS method are expressed as means.^9^ Correlation of true in vitro stock sample concentration versus measured concentration by UPLC‐MS/MS was checked using Bland‐Altman analysis.

Results were processed with Regress 10.01 in Microsoft Office Excel (version 2010; Microsoft Inc) and SPSS statistics (version 24.0.0.0; IBM).

## 3 RESULTS

### 3.1 Method validation

**Figure 1:**
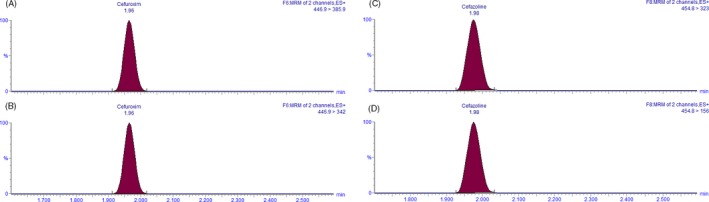
Chromatograms of cefuroxime and internal standard cefazolin in stock solution at LOQ (0.1 mg/L cefuroxime). The *X*‐axis shows retention time in minutes, and the *Y*‐Axis shows relative peak height in percentage. Cefuroxime and cefazolin eluted at 1.96 and 1.98 min, respectively. A, Cefuroxime recorded at *m/z* 446.9 > 385.9. B, Cefuroxime recorded at *m/z* 446.9 > 342. C, Cefazolin recorded at *m/z* 454.8 > 323. D, Cefazolin recorded at *m/z* 454.8 > 156

A representative chromatogram of cefuroxime and the internal standard cefazolin is shown in Figure 1. Cefuroxime and cefazolin eluted at 1.96 and 1.98 min, respectively. Cefuroxime plasma levels were tested for validation, specificity, linearity, repeatability, intermediate precision, recovery, and LOQ.

**Figure 2:**
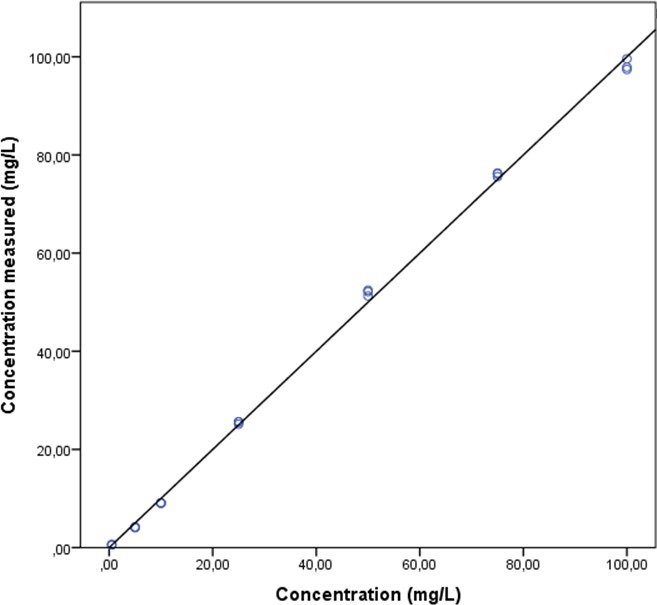
Linearity of UPLC‐MS/MS analysis of cefuroxime. Data are shown in triplicates as dots for a serial dilution of seven stock concentrations. Data represent measured concentrations. The real concentration of prepared stock solutions is shown along the *X*‐axis. The *Y*‐axis presents measured concentrations. A calibration line was calculated as follows: *Y* = *x*‐6E‐05 with *R* ^2^ = .9988 and *r* = .9994

**Figure 3:**
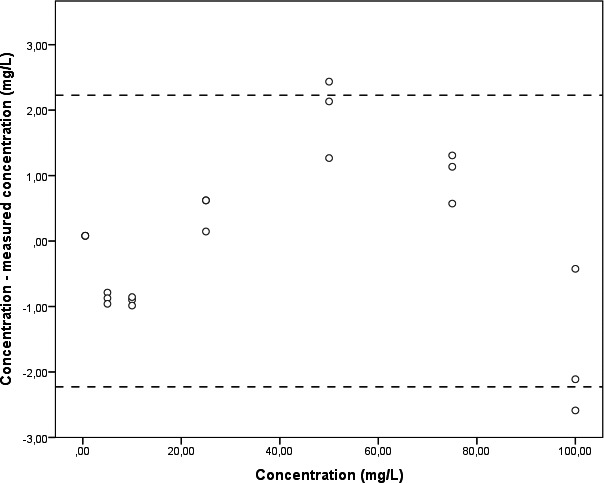
Bland‐Altman plot of the calibration line. Data representing measured concentrations relative to the real concentration of cefuroxime are shown as dots. Data are presented in triplicates for a serial dilution of seven stock concentrations. Dashed lines correspond to ± 1.96 standard deviations. The real concentration of prepared stock solutions is shown along the *X*‐axis. The *Y*‐axis presents the measured concentration of cefuroxime minus its real concentration

Linearity was confirmed in the 0.5‐100 mg/L range by correlation coefficient *r* = .9994 and regression coefficient *R* ^2^ = .9988. A calibration line was calculated as follows: *Y* = *x*‐6E‐05 (Figure 2). The corresponding Bland‐Altman plot is shown in Figure 3. Blank plasma relative to 0.5 mg/L cefuroxime gave 0.0% MRM transitions.

Repeatability expressed as intra‐assay coefficients of variation (CVs) was 6.18%‐2.59%. Intermediate precision expressed as inter‐assay CVs was 1.61%‐3.77%. Mean recovery was 99.7%‐100.3%. Finally, LOQ was 0.1 mg/L with a CV of 10.7%.

In vitro plasma samples spiked with cefuroxime at 2‐80 mg/L, corresponding to the clinical range, exhibited a mean plasma proteins‐bound cefuroxime fraction of 37.6% (range 34.26%‐41.48%).

### 3.2 Clinical application of the UPLC‐MS/MS method

Patient characteristics (N = 11) are shown in Table 1.

**Table 1:** Demographics and clinical variables of patients

	All patients (N = 11)
Age, median (range)	72 (54‐77)
Gender	
Male (N = 8)	72.7%
Female (N = 3)	27.3%
Actual body weight^a^ (kg), median (range)	81 (52‐113)
APACHE IV score	78 (59‐120)
eGFR (CKD‐EPI) (mL/min/1.73 m^2^), median (range)^a^	48.5 (7‐115)
Albumin serum concentration (N = 46^b^) (g/L)	19 (11‐40)
Renal replacement therapy	
IHD (N = 1)	9.1%
CVVH (N = 1)	9.1%
Sampling time (N = 18)	
Trough (N = 12)	66.7%
Mid (N = 3)	16.7%
Peak (N = 2)	11.1%
Continuous infusion (N = 1)	5.5%

a At the time of routine blood sampling used for cefuroxime quantification.

b Samples collected during whole ICU stay.

**Figure 4:**
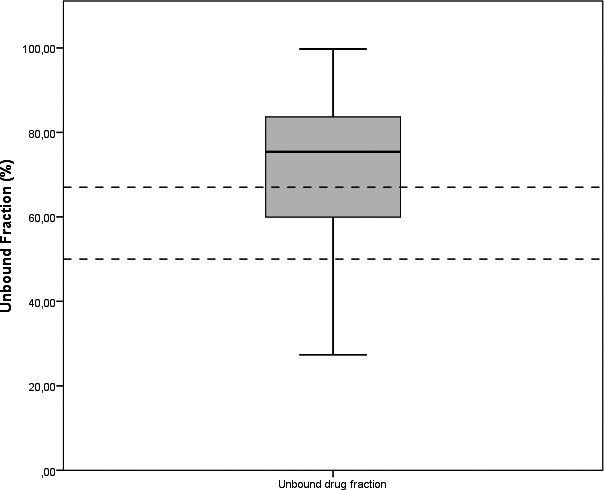
Box plot showing unbound cefuroxime fractions of all patients. Box plots showing interquartile ranges with median, minimum, and maximum fractions of cefuroxime presented as percentages. Standard unbound fraction (50%‐67%) published by the manufacturer 13 is shown by dashed lines. N = 18 measurements from N = 11 patients

We collected 18 usable leftover samples from 11 patients. Trough samples were collected from 9 patients. Three mid samples were collected from 3 patients. Two peak samples were collected from 2 patients and one continuous sample from 1 patient. Of some patients we collected multiple samples which differed in sampling time. Leftover samples used for cefuroxime quantification differed from day 1 of treatment to the last day of treatment. The longest treatment period was 22 days. Sampling timing varied from 126 min before administration to 312 min after administration. Median (range) renal clearance (eGFR) was 48.5 (7‐115) mL/min/1.73 m^2^. Figure 4 shows the unbound fraction of cefuroxime in our study population; the median was 75.42% (range 27.36%‐99.75%).

**Figure 5:**
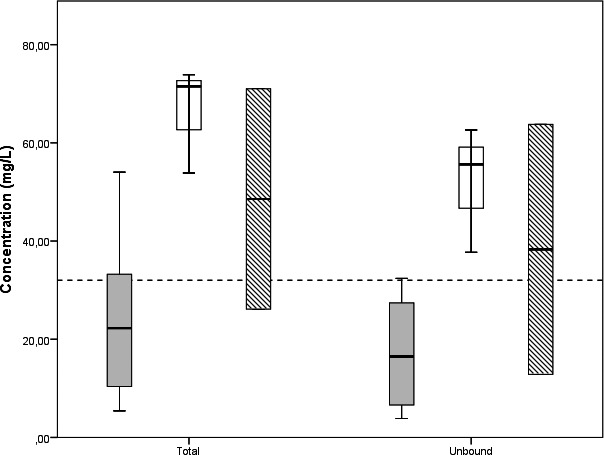
Box plots sorted by sampling time. Box plots showing interquartile ranges with median, minimum, and maximum concentrations of cefuroxime measured in the patient cohort. Intra‐individual difference in total and unbound concentration of cefuroxime is shown on the *X*‐axis. The *Y*‐axis represents the measured concentration of cefuroxime. The dashed line represents 4*MIC (32 mg/L). For clarity, only trough (plain grey), mid (striped), and peak (plain white) samples are shown. N = 12 trough samples, N = 3 mid samples, and N = 2 peak samples were obtained from 10 ICU patients

Figure 5 shows the concentration of total and unbound cefuroxime with respect to sampling time. The median trough concentration of total cefuroxime was 22.27 mg/L (range 5.42‐54.03 mg/L), with a corresponding median unbound concentration of 11.94 mg/L (range 3.85‐32.39 mg/L). Median mid concentration was 71.49 mg/L (range 53.87‐73.86 mg/L), with a corresponding unbound concentration of 55.62 mg/L (range 10.03‐62.62 mg/L). Two total peak concentrations were 71.01‐26.13 mg/L with corresponding unbound concentrations of 63.76‐12.85 mg/L. Continuous infusion resulted in a concentration of 40.82 mg/L, with an unbound concentration of 11.17 mg/L.

## 4 DISCUSSION

To our knowledge, this is the first method to quantify unbound cefuroxime plasma concentrations by UPLC‐MS/MS. The main finding of this prospective, noninterventional feasibility study is that UPLC‐MS/MS is suitable for rapid measurement of cefuroxime plasma levels within the therapeutic range of 0.5‐100 mg/L in a heterogeneous cohort of ICU patients. Precision and accuracy were within acceptable limits for clinical application in conformity with ISO 15189 requirements.^9^ By using an ultrafiltration device developed for protein filtration, we could detect similar protein‐bound fractions in in vitro spiked cefuroxime plasma samples as reported by the drug manufacturer.^13^ Unbound cefuroxime is responsible for its pharmacological activity. To optimize dosing strategies with cefuroxime treatment, analysis on unbound concentrations rather than total concentration is needed. We also showed that by using the unbound fraction instead of total cefuroxime concentration, most patients did not meet the required PK/PD target of 32 mg/L of cefuroxime, corresponding to four times the minimum inhibitory concentration (MIC).^14^ MIC values from EUCAST are established based on growth of bacteria in increasing cefuroxime concentrations which are not bound to proteins. Therefore, optimal target concentrations should be based on unbound cefuroxime concentrations. Several studies have recently shown that MS/MS is a reliable method for measuring cefuroxime in critically ill patients.^4^, ^14^, ^15^ We improved this method by adding and implementing a validated protocol for the quantification of unbound cefuroxime in a cohort of critically ill patients with low serum albumin levels and various stages of renal failure with dialysis dependency. The median unbound fraction in our population was 75.4%, which is substantially more than the 50%‐67% published by the manufacturer based on healthy volunteers.

Our results are in line with previous studies showing that hospitalized patients present lower protein binding and thus a higher unbound fraction of cefuroxime.^16^ Unbound plasma concentrations of cefuroxime cannot be accurately predicted when they are extrapolated using standard plasma protein binding (33%‐50%) on in vivo total concentrations, especially in ICU patient samples.^2^ Low protein binding in ICU patients is caused by reduced albumin binding and competition with endogenous substrates, such as urea and bilirubin, that accumulate due to reduced renal clearance.^17^, ^18^, ^19^ An increase in volume of distribution by fluid resuscitation in septic patients may also account for higher unbound fractions of beta‐lactam antibiotics.^20^

A major strength of our study was the inclusion of a heterogeneous group of ICU patients, and blood samples were taken at random times. All ICU patients underwent standard care, making our results generalizable to other compatible ICU cohorts. Our study refines the current use of MS/MS in ICU patients, illustrating its potential to increase routine TDM for cefuroxime.^14^, ^15^, ^16^, ^17^

There are also limitations of our study. First, instead of applying a standard research protocol, we used leftover samples. Second, this study was restricted to adult ICU patients recovering from a mixture of medical‐surgical procedures, making generalizability of our finding to other cohorts of ICU patients cumbersome. Third, we could only measure total protein‐bound quantities but could not differentiate between specific proteins, such as immunoglobulins, proteins originated from total parenteral nutrition, or others. However, this limitation is of lesser clinical relevance, as we were mainly interested in the relative quantity of unbound cefuroxime required to reach target PK/PD levels. Finally, we used cefazolin as an internal standard and that might have prompted interference because cefazolin is widely used as prophylactic treatment for surgery. However, in our hospital, the pharmacists who interpret cefuroxime concentrations are aware of all the medications prescribed and administered to each patient during hospital stay. Medication prescriptions and administrations are digitally recorded in the patient data management system. This is the case for all hospitals in the Netherlands. Secondly, cefuroxime and cefazolin are not used simultaneously as defined in our local antibiotic treatment guideline, also cefazolin is quickly cleared (*t*½ = 1.5‐2 hours). Therefore, using cefazolin as an internal standard will be not a problem for clinical practice.

In summary, we describe a simple and sensitive UPLC‐MS/MS method for the quantification of cefuroxime in plasma obtained from ICU patients. Due to its high sensitivity and accuracy, this method allows pharmacokinetic analysis and TDM‐based calculation of unbound cefuroxime plasma levels with new dosage regimens, which is especially relevant for ICU patients with hypoalbuminemia and renal failure.

By measuring unbound fractions of cefuroxime our method could improve the treatment of ICU patients, for whom achieving correct and effective treatment as fast as possible is of special importance.^2^ The method was implemented in our hospital. UPLC‐MS/MS gives a quick output of results, so dosing strategies can be adjusted in real time based on TDM of unbound plasma levels rather than total concentrations. TDM on unbound fractions of antibiotics can become common practice for improving in‐hospital antibiotic treatment. Future research within our hospital will focus on TDM of antibiotics in patients with high risk of altered pharmacokinetics.
